# CrFeVW*X* (*X* = Ta or Ti) High-Entropy Alloy: A Theoretical and Experimental Comparative Investigation on Phase Stability

**DOI:** 10.3390/ma19050987

**Published:** 2026-03-04

**Authors:** Ricardo Martins, Vasco Valadares, André Pereira, António P. Gonçalves, Filipe Neves, Ana Sá, Paulo Luz, Bernardo Monteiro, Andrei Galatanu, Judith Monnier, Benjamin Villeroy, Marta Dias

**Affiliations:** 1 Instituto de Plasmas e Fusão Nuclear (IPFN), Instituto Superior Técnico, Universidade de Lisboa, Av. Rovisco Pais, 1049-001 Lisboa, Portugal; vascovaladares@tecnico.ulisboa.pt (V.V.); andre.sampaio.pereira@tecnico.ulisboa.pt (A.P.); bernardo.monteiro@ctn.tecnico.ulisboa.pt (B.M.); marta.dias@ctn.tecnico.ulisboa.pt (M.D.); 2 Centro de Física e Engenharia de Materiais Avançados (CeFEMA), Instituto Superior Técnico, Universidade de Lisboa, Estrada Nacional 10, 2695-066 Bobadela, Portugal; apg@ctn.tecnico.ulisboa.pt; 3 Departamento de Engenharia e Ciências Nucleares (DECN), Instituto Superior Técnico, Universidade de Lisboa, Estrada Nacional 10, 2695-066 Bobadela, Portugal; 4 Laboratório Nacional de Energia e Geologia (LNEG), Estrada do Paço do Lumiar, 1649-038 Lisboa, Portugal; filipe.neves@lneg.pt (F.N.); ana.sa@lneg.pt (A.S.); paulo.luz@lneg.pt (P.L.); 5 National Institute of Materials Physics, Atomistilor Street 405 A, 077125 Magurele, Romania; gala@infim.ro; 6 Université Paris Est Créteil, CNRS, ICMPE, UMR 7182, 2 rue Henri Dunant, 94320 Thiais, France; judith.monnier@cnrs.fr (J.M.); benjamin.villeroy@cnrs.fr (B.V.)

**Keywords:** high-entropy alloys, Molecular dynamics, Monte Carlo simulation, microstructure, Mechanical alloying parameters

## Abstract

Materials capable of withstanding extreme environments open promising opportunities for nuclear fusion reactors. In this study, equiatomic CrFeTaVW and CrFeTiVW high-entropy alloys are investigated as interlayer materials between W and CuCrZr. Monte Carlo and Molecular Dynamics simulations predicted a bcc-type structure for both systems. Additionally, the Monte Carlo simulation predicts lower potential energy and a more stable structure for both systems than Molecular Dynamics. For CrFeTaVW, the chemical segregation values are lower in MC than in the MD simulation, whereas for CrFeTiVW, the opposite trend is observed, with MC indicating stronger segregation values. After simulation, the high-entropy alloys were prepared by planetary ball milling, consolidated by spark plasma sintering, and analyzed using X-ray diffraction, scanning electron microscopy, and thermal diffusivity. The experimental results for the milled powders confirmed the formation of a bcc structure in both alloys. The consolidated material revealed a bcc-type structure and an Fe_2_Ta Laves phase for the CrFeTaVW HEA, while the CrFeTiVW HEA exhibits two different bcc-type structures. The values of CrFeTaVW and CrFeTiVW thermal diffusivity are between 3.5 and 7 mm^2^/s, which is consistent with the expected values for high-entropy alloys. Overall, the findings indicate that these HEAs have promising properties that can be used in extreme environments.

## 1. Introduction

Selecting suitable materials for nuclear reactors is critical for efficient and safe energy production. One of the main challenges is managing the extreme heat generated during operation, without compromising structural integrity. Tungsten (W) has been chosen for plasma facing components [1,2] in the divertor, owing to its high melting point [3], exceptional thermal resistance [4], low sputtering yield, and low tritium retention [5]. However, its application is limited by a relatively high ductile-to-brittle transition temperature (DBTT) [6]. To dissipate the heat absorbed by plasma facing components, a CuCrZr alloy has been employed as a heat sink [7]. Despite its favorable thermal conductivity, this alloy is prone to embrittlement under irradiation [8] and has a relatively narrow operational temperature range [9]. As a result, an intermediate layer is necessary to ensure efficient thermal transfer between W and CuCrZr, while maintaining each material within its optimal temperature range.

Unlike traditional alloys, high-entropy alloys (HEAs) typically consist of five or more principal elements and often form simple and stable single-phase solid solutions such as face-centered cubic (fcc) [10], body-centered cubic (bcc) [11], and hexagonal close-packed (hcp) [12] structures. Their stability is mainly attributed to their high configurational entropy, which lowers the Gibbs free energy and promotes solid solution formation [13,14]. HEAs have demonstrated superior mechanical and thermal properties compared to conventional alloys [15]. Notably, some compositions, such as MoNbTaW and MoNbTaVW, exhibit excellent strength retention at elevated temperatures [16], while others, including Ni-based HEAs like Mo_6.6_Nb_6_Ni_80_Ta_1.4_Ti_6_, show exceptional thermal stability [17]. Studies show that increasing Ti content in the CoCrFeNiMnTi_x_ HEA alters its structure from fcc to bcc [18], thereby improving its mechanical strength. However, excessive Ti content can reduce tensile strength. Karimzadeh, M. [19] reports a (CoCrFeNi)_(1−*x*)_Ti*_x_* where an increase in titanium content results in an increase in the shear strength and shear elongation due to grain refinement [19]. Additionally, in the Al_24.5_Fe_31_Mn_28_Ni_15_Ti*_x_* HEAs [20], Ti increases hardness through solid solution strengthening and reduced lamellar spacing, although excessive heat treatment can diminish this benefit [20]. Huo, Wenyi [21] investigated a tantalum-based HEA, CoCrFeNiTa*_x_* (*x* = 0.1–0.5 molar ratio), as a potential structural material for critical load-bearing applications. These alloys consist of an fcc-type-structure solid solution with a Laves phase. Increasing Ta content shifts the solidification from hypoeutectic to hypereutectic, yielding an ultrahigh yield strength of 1.4 GPa for *x* = 0.395. In contrast, the *x* = 0.3 hypoeutectic alloy exhibits a compressive strength of 2.5 GPa and a fracture strain of 44% [21], meaning that lower Ta content induced a higher yield strength with good ductility. Furthermore, Ren, Faling [22] studied NbTaTiV HEA, which demonstrates a yield strength (σᵧ) of 770 ± 16 MPa and an ultimate tensile strength (σuts) of 835 ± 11 MPa, while HfNbTaTiZr exhibits higher strength values (σᵧ = 992 ± 21 MPa; σuts = 1064 ± 20 MPa). Guo et al. [23], TiVNbTa RHEA reported exceptional ~40% strain combined with a high yield strength of around 800 MPa. The authors show that a single-phase BCC with a dendritic microstructure can retain a yield strength of 330 MPa and a compressive strength of 423 MPa at 30% strain [23] at elevated temperature (1273 K), demonstrating good high-temperature mechanical stability. These results highlight a favorable balance between strength, ductility, and stability at high temperatures, depending strongly on composition and phase constitution.

Alongside experimental characterization, simulations provide a powerful tool for probing and understanding the behavior of HEAs. Molecular Dynamics (MD) simulations are a tool to investigate these materials by modeling atomic interactions and dynamic behavior under various conditions. In MD, atoms are represented as point masses that follow Newton’s laws of motion [24]. The Metropolis–Monte Carlo (MC) method will complement the MD by enabling atom swaps to explore more stable configurations. The hybrid MD-MC approach is an efficient method for simulating HEAs [16]. LAMMPS software supports both methods and can also simulate X-ray diffraction (XRD) patterns based on atomic configurations [25,26]. Interatomic forces are described using the Embedded Atom Method (EAM) potential, which relates the total energy to the local electron density, providing accurate modeling of metallic systems [24,27,28]. This approach has been demonstrated to be really useful in previous works such as [29,30,31].

In this study, the simulation, development, and characterization of equiatomic CrFeTaVW and CrFeTiVW high-entropy alloys are presented. Structural characterization was performed using scanning electron microscopy (SEM) and X-ray diffraction (XRD). Thermal properties of the alloys were evaluated and are discussed.

## 2. Materials and Methods

Equiatomic CrFeTaVW and CrFeTiVW alloys were simulated using periodic boundary conditions. For bcc simulations, the number of atoms was 4394 (13^3^ cells), and for fcc simulations, the number of atoms was 4000 (10^3^ cells). The typical simulation sequence involved several steps of energy minimization of the initial configuration, followed by heating to the simulation temperature and energy minimization for 6 × 10^6^ time steps. The hybrid Molecular Dynamics/Monte Carlo simulation consisted of introducing a Monte Carlo swap attempt every 10 Molecular Dynamics simulation steps. Atomic-swap operations (MC attempts) were performed on randomly chosen pairs of different types of atoms. The temperature used in the Metropolis criterion, which dictates swap probabilities, was the same as that of the isothermal MD simulation. Pressure was maintained at 1 atmosphere; the simulation used an isothermal–isobaric ensemble (NPT) in LAMMPS, see Support Information, LAMMPS inputs. The same procedure, using the same data set for simulation of a binary alloy, has been reported in the literature [31,32], as well as other HEAs, such as FeTaTiW*A*, where *A* = Cu, V, or Cr [29,30,31]. The software LAMMPS from Sandia Labs [33] was used for (i) Molecular Dynamics (MD), (ii) hybrid Molecular Dynamics/Monte Carlo simulations (MC), and (iii) X-ray diffraction calculation of MD and MC simulated materials. Regarding potentials, the usual EAM potential [34] was mainly used, but as this does not include Cr and V, it was completed with an MIE potential table [35] for interactions involving these elements. Chemical segregation was quantified using the standard deviation of local atomic concentrations. The simulation cell was divided into 27 spatial sampling regions. Within each region, atoms inside a spherical volume of 9 nm diameter were used to calculate the local composition. The local concentrations were averaged over N simulation steps. The standard deviation was then determined from the variation of the averaged local concentrations relative to the overall mean concentration of the system. A higher standard deviation indicates stronger chemical segregation, while a lower value corresponds to a more homogeneous atomic distribution [36].

Powders of Cr, Fe, Ta, Ti, V, and W (AlfaAesar, 99.9% nominal purity with an average particle size of 10 µm) were mixed in an equiatomic proportion in a glove box and mechanically alloyed using a high-energy planetary ball mill, Retsch PM 400 MA (Retsch GmbH, Haan, Germany), with WC balls and vials. The ball-to-powder mass ratio was 10:1, and milling was performed at 350 rpm (rotations per minute) for 2 h.

Powder X-ray diffraction (PXRD) was used to investigate the evolution of the powder mixtures using a Philips X’Pert diffractometer in Bragg–Brentano geometry with Cu Kα radiation, over a 2θ range of 10° to 80°, with a 0.02° 2θ step size and 2 s per step. The microstructures were detected in backscattered electron (BSE) and secondary electron (SE) imaging modes with a Thermo Scientific Phenom ProX G6 scanning electron microscope(Thermo Fisher Scientific Inc., Waltham, MA, USA), equipped with a 15 keV electron beam.

The consolidation of the powders was performed by spark plasma sintering (SPS) in collaboration with Romania, using an German FCT System GmbH (FCT System GmbH, Kuppenheim, Germany) sintering machine at a pressure of 70 MPa and a temperature of 1543 K for a holding time of 5 min, and in collaboration with France, using the Dr. Sinter 515S Syntex setup belonging to the “Plateforme de Frittage Ile de France” (Thiais) with the following conditions—under vacuum, graphite die, diameter 10 mm, pressure 80 MPa at 1573 K—for CrFeTaVW.

Thermal diffusivity measurements were made using the laser flash method (LZT system, Linseis, Massegarate, Germany). The specimen was subjected to a short-duration laser pulse (0.5 ms) at 300 V, under a helium atmosphere maintained at 0.05 bar at room temperature with a heating rate of 5 °C min^−1^.

Thermodynamic calculations were first performed to determine the possible structures formed in the alloys and to aid in the interpretation and discussion of the results. Based on empirical models [37,38] using the enthalpies and entropies of mixing, Δ*H_mix_* and Δ*S_mix_*, the atomic size differences, δ, and the valence electron concentrations, VEC, it is possible to predict the formation of solid solutions in the ranges −15 kJ/mol ≤ Δ*H_mi__x_* ≤ 5 kJ/mol, 11 J/(K·mol) ≤ Δ*S_mix_* ≤ 19.5 J/(K·mol), and 1 ≤ δ ≤ 6.5. The most stable phases predicted are bcc for VEC < 6.87, and fcc for VEC ≥ 8. Between these values, mixed fcc and bcc structures are expected to coexist. It should be noted that the Miedema model, while widely used for estimating mixing enthalpy in HEAs, has limitations for accurate prediction in complex multicomponent systems, sometimes due to not accounting for vibration entropy [39]. Therefore, it is considered a trend-based thermodynamic insight, and it should not be interpreted as an exact quantitative predictor. In this context, calculations of the relevant properties of the alloy are presented in Table 1. Based on the δ calculated values, both (CrFeTaVW) and (CrFeTiVW) systems display lower values than 6.5, displaying a bcc-type-structure solid solution.

## 3. Results

### 3.1. Simulation

**Figure 1:**
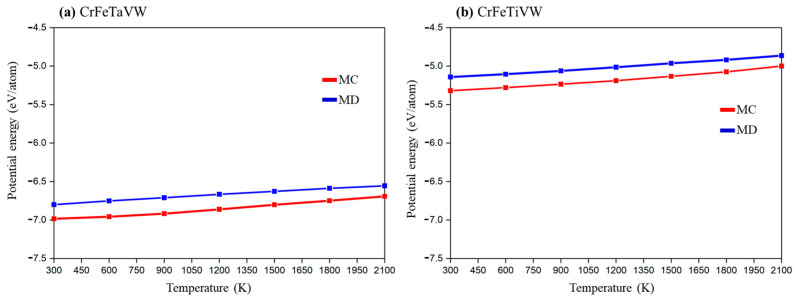
Potential energy versus temperature for Monte Carlo (MC) and Molecular Dynamics (MD) simulations of the (**a**) CrFeTaVW and (**b**) CrFeTiVW HEAs.

The temperature dependence of the potential energy for CrFeTaVW and CrFeTiVW HEAs is shown in Figure 1a and Figure 1b, respectively. In both systems, the MD and MC simulations of potential energy as a function of temperature display similar trends, with a slight but constant deviation across the temperature range. Notably, the MC simulation yields lower potential energy values, suggesting a more stable structure. A comparison between CrFeTaVW and CrFeTiVW shows that the CrFeTaVW HEA has a considerably lower potential energy than the CrFeTiVW HEA, which suggests a more stable high-entropy alloy, which might be associated with the lower atomic size difference displayed in Table 1.

**Figure 2:**
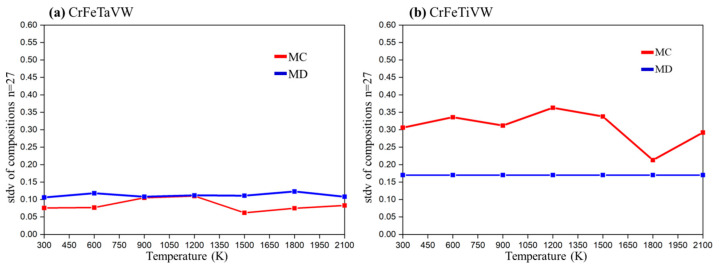
Chemical segregation as a function of temperature for Monte Carlo (MC) and Molecular Dynamics (MD) simulations for (**a**) CrFeTaVW and (**b**) CrFeTiVW HEAs.

The standard deviation values (SDVs) of the chemical segregation as a function of temperature for CrFeTaVW and CrFeTiVW HEAs are presented in Figure 2a and Figure 2b, respectively.

For CrFeTaVW (Figure 2a), the MD simulation (identified by the blue marker) yields no change in chemical segregation standard deviation values as temperature increases, generating a random distribution of the elements (uniform solid solution). In contrast, the MC simulations (identified by the red marker) show no chemical segregation until 600 K. Upon reaching 600 K and continuing to 1500 K, a higher SDV is identified, indicating a higher degree of segregation, eventually reaching values similar to those obtained in MD simulations. At 1500 K, the segregation values decrease to the initial values, producing a uniform random distribution of the elements. The results suggest a temperature range between 600 K and 1500 K where segregation is most likely to occur.

For CrFeTiVW, shown in Figure 2b, both MD and MC simulations show distinct trends. MD simulations exhibit a constant standard deviation across the entire temperature range, consistently lower than that of MC simulations. MC simulation indicates a slight increase in standard deviation from the initial temperature to 1200 K. Beyond 1200 K, there is a small reduction up to 1500 K, followed by a sharp reduction to 1800 K, reaching the lowest SDV observed for MC simulation. From 1800 K to 2100 K, chemical segregation increases, reaching a similar SDV to that at 300 K. The MC simulation predicts some element segregation from 300 K to 1500 K. The highest segregation value is found at 1200 K, indicating a higher probability of segregation at this temperature, even though the SDV increases only slightly from 300 K to 1200 K.

**Figure 3:**
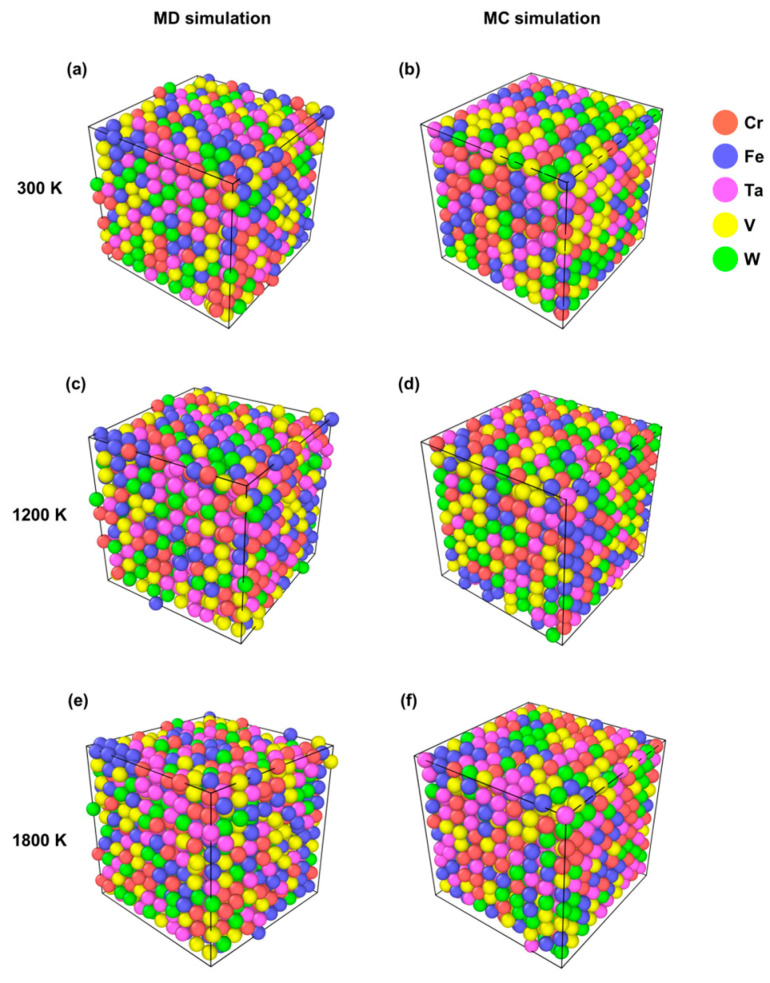
Representation of the structures simulated for MD and MC structures for CrFeTaVW at (**a**,**b**) 300 K, (**c**,**d**) 1200 K, and (**e**,**f**) 1800 K, respectively.

Figure 3a and Figure 3b show the MD and MC simulated structures, respectively, at 300 K obtained for the CrFeTaVW HEA, which display a random distribution of the five elements, similar to that of the starting structure, while Figure 3c,d display the simulation at T = 1200 K, and Figure 3e,f show the simulation at 1800 K. The results show no evidence of structural segregation in either MD or MC simulations, since it is not observed in localized clusters of the same element within each bcc structure. The simulation for the CrFeTaVW shows a bcc-type structure, and the lattice parameters obtained were *a* = 0.306 nm for MC and MD at 300 K.

**Figure 4:**
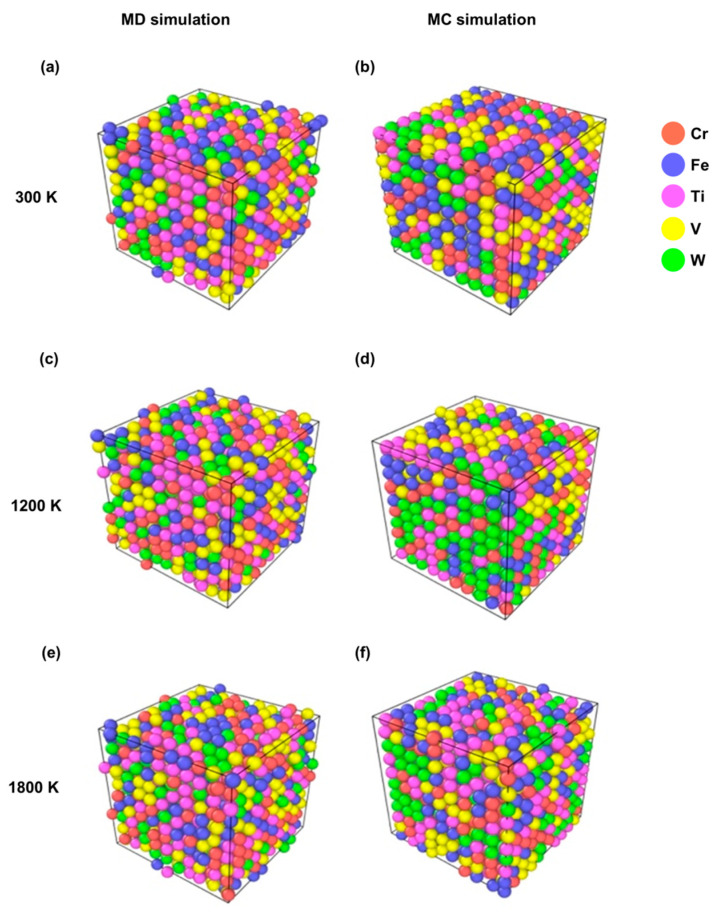
Representation of the structures simulated for MD and MC structures for CrFeTiVW at (**a**,**b**) 300 K, (**c**,**d**) 1200 K, and (**e**,**f**) 1800 K, respectively.

Figure 4a and Figure 4b show the MD and MC structures, respectively, obtained for the CrFeTiVW simulation at 300 K, in which the random distribution of the five elements is similar to that of the initial structure. In contrast, for the structure simulation at 1200 K, Figure 4c,d show limited tungsten segregation in the MC simulation but not in the MD simulation, as indicated by the small clusters of green atoms in Figure 4d. At 1800 K, as displayed in Figure 4e,f, there is no structure segregation resulting from either MC or MD simulations. The CrFeTiVW simulation shows a bcc-type structure, and the lattice parameters extracted were *a* = 0.313 nm for MC and *a* = 0.307 nm for MD at 300 K.

**Figure 5:**
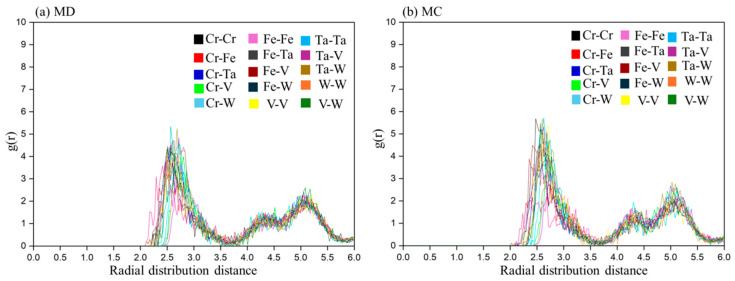
Simulation results at 300 K for (**a**) MD and (**b**) MC pair distribution functions for CrFeTaVW alloy.

The CrFeTaVW partial radial distribution function (RDF) as a function of temperature for all element bond types, corresponding to MD and MC simulations at 300 K, is presented in Figure 5. Looking at the RDF, a scaled histogram of the probability of each interatomic distance in the material shows a well-defined series of peaks in the pair distribution function, indicating a bcc crystal lattice. The first set of major peaks represents the distance to the eight nearest neighbors, while subsequent, smaller peaks correspond to next-nearest neighbors [40].

Regarding a single element, the most evident feature from the analysis of the first near neighbors (NNs) of all elements is that no segregation is perceived (very low probability of element bonding). At lower temperatures, the MC simulation yields a lower potential energy (Ep) corresponding to a more stable structure than MD. In both types of simulations, the crystal structure obtained was fully bcc.

**Figure 6:**
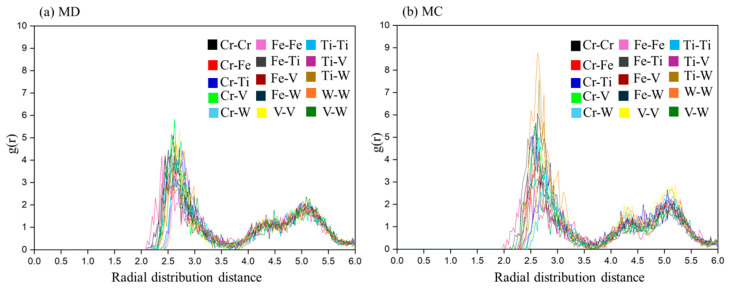
Simulation results at 300 K for (**a**) MD and (**b**) MC pair distribution functions for CrFeTiVW alloy.

The CrFeTiVW HEA partial radial distribution function (RDF) for MD and MC simulations as a function of temperature is shown in Figure 6a and Figure 6b, respectively. Although the MC simulation yields a lower potential energy (Ep) than the MD simulation, corresponding to the more stable structure shown in Figure 1, at lower temperatures than in MD, no segregation is observed (with a very low probability of N-N bond formation). However, the MC simulation, as shown in Figure 6b, displays a higher probability of a W-W bond.

### 3.2. Structural Analysis

**Figure 7:**
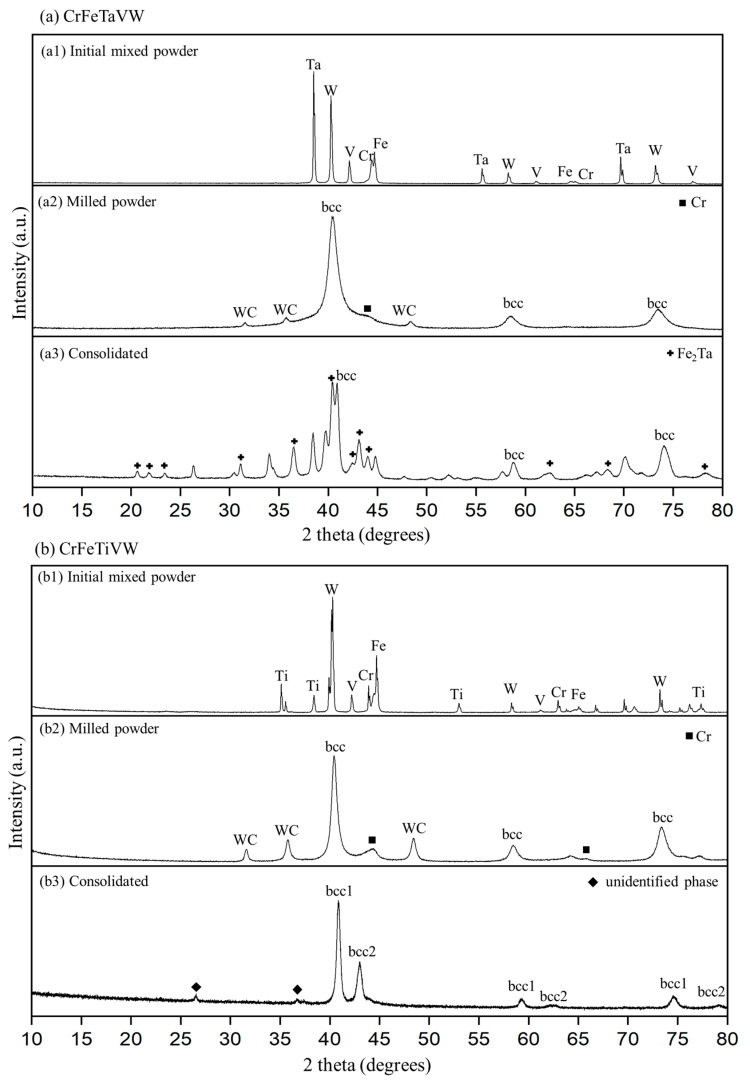
Experimental diffractogram for CrFeTaVW and CrFeTiVW of (**a1**,**b1**) the mixture of the initial powder, (**a2**,**b2**) the milled powder, and (**a3**,**b3**) the consolidated HEA. Pure chromium is indicated in the figure with a black square marker, and the unidentified phase is indicated as a black diamond.

Figure 7 presents the experimental diffraction patterns obtained at different processing stages for the CrFeTaVW and CrFeTiVW high-entropy alloys. Figure 7(a1–a3) correspond to the experimental diffractogram for the CrFeTaVW powder before milling and after milling and the consolidated CrFeTaVW HEA, respectively. Similarly, Figure 7(b1–b3) show the same processing sequence for the CrFeTiVW alloy system.

A comparison between the elemental mixture without milling in Figure 7(a1) and the milled powder in Figure 7(a2) reveals that the characteristic individual elemental peaks disappeared, and a body-centered cubic (bcc)-type structure with a lattice parameter of *a* = 0.315 nm was identified. Analogous behavior is found for the CrFeTiVW alloy, as shown in Figure 7(b1,b2). The bcc-type structure peaks shown in Figure 7(b2) have a lattice parameter of *a* = 0.315 nm. Peaks associated with the presence of WC are perceived in both systems, suggesting erosion of the milling spheres and the containers.

Additionally, low-intensity peaks corresponding to residual unmixed chromium (highlighted by black squares) are observed in both systems at the milled stage. The experimental diffractogram of the consolidated CrFeTaVW alloy, shown in Figure 7(a3), shows a persistent bcc-type structure phase with a lattice parameter *a* = 0.312 nm. Additional reflections (marked with a black cross) were attributed to an Fe_2_Ta intermetallic C14 Laves phase. As expected, the Cr phase exhibited in the milled state was suppressed upon sintering, either due to Cr dissolution in the alloy solid solution under sintering conditions or due to its incorporation into the Laves phases. The formation of the Fe-Ta Laves phase in the consolidated CrFeTaVW alloy can be attributed to the strong negative enthalpy of mixing combined with their significant atomic size difference. These factors promote the thermodynamic stability of intermetallic compounds of the AB_2_ type during consolidation. However, several minor peaks remained unindexed, implying the presence of secondary phases or structural variations not reported in the literature.

For the sintered CrFeTiVW alloy (Figure 7(b3)), the diffraction pattern reveals the formation of two distinct bcc-type structures. The first, denoted as bcc1, has a lattice parameter of *a* = 0.310 nm, while the second, bcc2, has a lattice parameter of *a* = 0.298 nm, as well as a small number of peaks that could not be identified. These results agree with the simulation experiments, which predicted two bcc-type structures at 1800 K: *a* = 0.307 nm by MD and *a* = 0.313 nm by MC.

**Figure 8:**
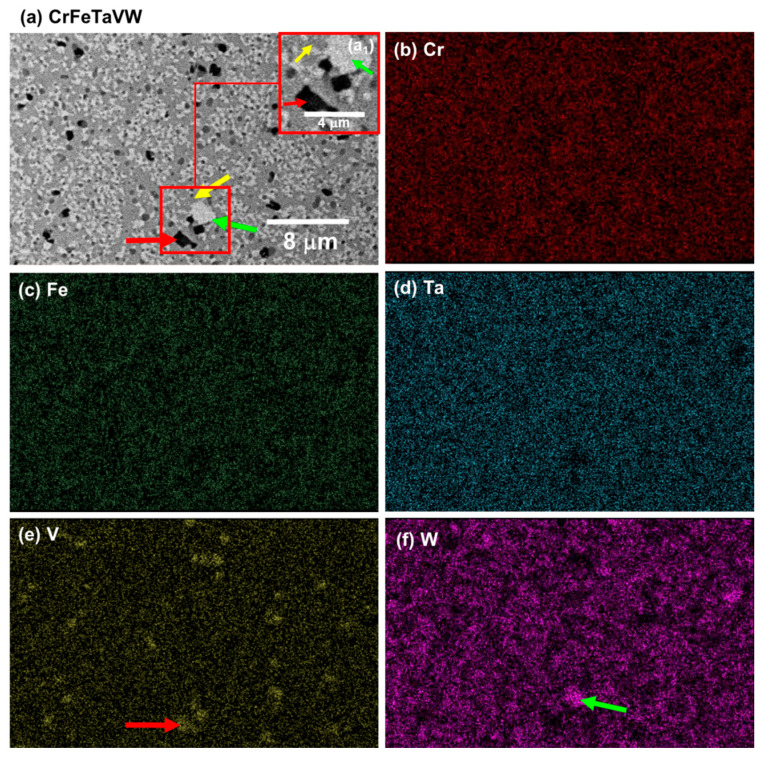
SEM images of the (**a**) consolidated CrFeTaVW high-entropy alloy; corresponding EDS elemental maps of (**b**) Cr-Kα; (**c**) Fe-Kα; (**d**) Ta-Lα; (**e**) V-Lα; and (**f**) W-Lα X-ray lines. (**a1**) Area magnification. The yellow arrow indicates the major phase; (**a**,**e**) the red arrow highlights the black phase (Ti-rich); (**a**,**f**) the green arrow indicates the light phase (W-rich).

**Figure 9:**
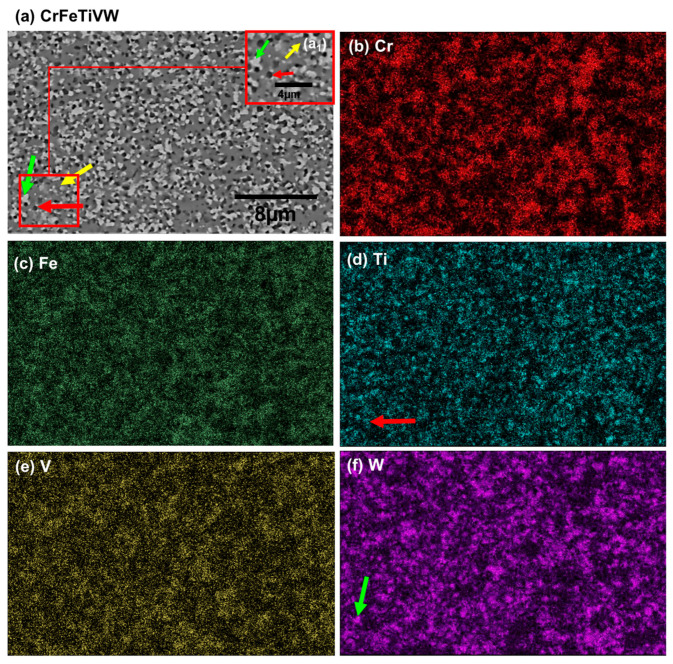
SEM images of the (**a**) consolidated CrFeTiVW high-entropy alloy; corresponding EDS elemental maps of (**b**) Cr-Kα; (**c**) Fe-Kα; (**d**) Ta-Lα; (**e**) V-Lα; and (**f**) W-Lα X-ray lines. (**a1**) Area magnification. The yellow arrow indicates the major phase; (**a**,**d**) the red arrow highlights the black phase (Ti-rich); (**a**,**f**) the green arrow highlights the light phase (W-rich).

Figure 8a and Figure 9a display scanning electron microscopy (SEM) images of the as-sintered CrFeTaVW and CrFeTiVW alloys, along with the corresponding energy-dispersive spectroscopy (EDS) elemental maps. Both sintered alloys exhibited submicrometric phases and a dense microstructure (>98% densification) with no visible porosity.

The microstructure of the CrFeTaVW HEA displayed in Figure 8a shows submicrometric phases with three distinct features that were identified in the microstructure. The lighter-contrast phase (identified by the green arrow) is a W-rich phase, while the intermediary regions (identified by the yellow arrow) correspond to the primary phase and comprise all elements. The darker grey regions (identified by the red arrow) are rich in V. Additionally, it is possible to observe that Cr, Fe, and Ta appear to be homogeneously distributed. The combination of SEM imaging with the EDS mapping confirms the segregation of V and W within the alloy. The consolidated CrFeTiVW HEA, as shown in Figure 9, also exhibits a submicrometric microstructure with three distinctive phases. The lighter-contrast phase (identified by the green arrow) is rich in W, while the intermediary regions (identified by the yellow arrow) correspond to the major phase, which comprises all elements where minor Ti and W areas and Cr-, Fe-, and V-rich areas are detected. The darker grey regions (identified by the red arrow) are rich in Ti.

The simulation, together with the experimental results, allows us to clarify that for the CrFeTiVW alloy, MC simulations point to a pronounced chemical segregation in the temperature range between 300 and 1500 K, as reflected by increased standard deviation values of the local composition (Figure 2). This trend is further supported by the pair distribution function analysis, which shows an increased probability of W–W bonding. Experimentally, the consolidated microstructure reveals Ti- and W-rich regions (Figure 8), possibly confirming the segregation tendency predicted by the MC simulations. In contrast, no significant segregation was predicted for CrFeTaVW in the simulations. However, experimental observations show the Cr-, Fe- and V-rich phase regions and the Ti-rich and W-rich regions observed in Figure 9. The discrepancy between simulation and experiment can be attributed to kinetic effects not captured by the simulation approach. The MC method employs atom swaps governed purely by thermodynamic energy minimization and does not account for diffusion kinetics. Therefore, the phases observed in the experimental part might not be fully reproduced by the simulations. Overall, the experimental results align more closely with the thermodynamically driven MC predictions under sintering conditions, while deviations can be explained by kinetic limitations, diffusion constraints, cooling rate, and non-equilibrium processing effects. These comparisons validate the predictive capability of the computational approach in describing thermodynamically favored segregation tendencies. Therefore, the results should be interpreted as predictive indicators rather than definitive experimental confirmation.

Since the SPS process involves very rapid heating rates and short sintering times, it may introduce kinetic constraints and non-equilibrium compositional inhomogeneities. Such rapid processing can limit long-range diffusion and promote localized elemental segregation that is not solely governed by thermodynamic equilibrium. Since simulations primarily describe thermodynamically driven atomic rearrangements, the Monte Carlo swaps are controlled by the Metropolis criteria and are not explicitly governed by real diffusion kinetics. The simulations provide insight into equilibrium segregation tendencies and should thus be interpreted as qualitative rather than strictly quantitative.

The CrFeTaVW microstructure shown in Figure 8a reveals the presence of three distinct phases. One major matrix phase contains all constituent elements, with Cr, Fe, and Ta homogeneously distributed while V and W are presented in lower concentrations. In addition, two secondary matrices are observed: a V-rich phase corresponding to the darker-contrast regions, and a W-rich phase corresponding to the lighter-contrast regions. The Cr-, Fe- and Ta-rich phase can be explained by the formation of AB_2_ stoichiometric compounds, where the bigger atomic radius atoms (such as W, Ti, Mo, Zr, and Ta) occupy the A sites while the smaller atoms (such as Cr, Fe, and V) occupy the B sites [41], forming AB_2_ Laves phases similar to the ones reported in the literature [41]. Furthermore, the W- and Ti-rich phases are likely associated with bcc crystal structures, as indicated by the experimental diffractogram shown in Figure 7(a3). Regarding the CrFeTiVW HEA, the experimental diffractogram exhibited in (Figure 7(b3)) reveals two bcc solid solution phases. These phases can be correlated with the three distinct compositional regions observed in the microstructure (Figure 7(a3)). The W-rich and Ti-rich regions can be indexed to the bcc1 structure, while the Cr-, Fe- and V-rich regions can be indexed to the bcc2 peaks in agreement with the literature [42].

### 3.3. Thermal Diffusivity

**Figure 10:**
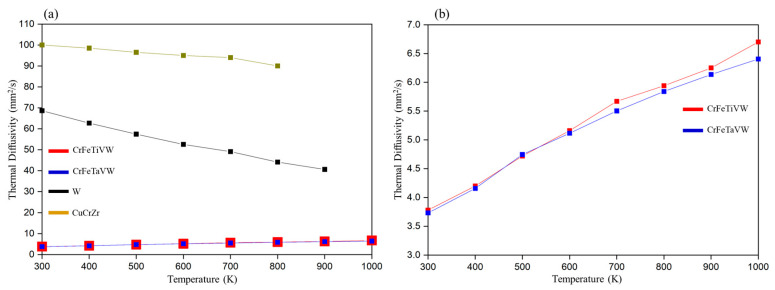
(**a**) Thermal diffusivity of consolidated CrFeTaVW and CrFeTiVW samples together with CuCrZr [44,45] and pure W [48], and (**b**) a magnification with only the thermal diffusivity for both samples.

Figure 10 illustrates the thermal diffusivity of the CrFeTaVW and CrFeTiVW high-entropy alloys over the range of 300 K to 1000 K. Both samples exhibit similar thermal diffusivity behavior as a function of temperature, with diffusivity values increasing as the temperature rises and reaching between 3.5 mm^2^/s and 7 mm^2^/s. The change from Ta to Ti does not produce any major alteration in the thermal diffusivity values. These values are within the range (3–15 mm^2^/s) observed in previous works FeTaTiVW [29] and (CrFeTaTi)_70_W_30_ [30]. Furthermore, compared with pure tungsten [43] and CuCrZr [44,45], both CrFeTaVW and CrFeTiVW high-entropy alloys exhibit significantly lower diffusivity values. In high-entropy alloys, atomic-scale disorder shortens the phonon mean free path, resulting in high phonon scattering rates. According to Lu, Chieh Lien [46], in HEAs, the increase in thermal diffusivity at high temperatures is driven by lattice expansion, which lengthens the phonon mean free path as temperature rises. Since tantalum and titanium have nearly identical atomic radii (0.143 nm and 0.146 nm) [47], substituting one for the other causes little lattice disruption. Consequently, the thermal diffusivity remains essentially unaffected.

Overall, the results suggest that substituting Ta with Ti in the CrFeTaVW alloy to form CrFeTiVW has only a minor effect on the microstructure, showing parallel microstructural features. However, their experimental diffractograms reveal notable differences. CrFeTaVW XRD shows a bcc-type structure with a lattice parameter of *a* = 0.312 nm, along with a C14 Laves phase. In contrast, CrFeTiVW displays two distinct bcc-type structures. The first, denominated bcc1, has a lattice parameter *a* = 0.310 nm, and the second, bcc2, has *a* = 0.298 nm. Moreover, no significant differences in thermal diffusivity were observed between the two alloys. Based on the results, the only influence of Ti and Ta on CrFeVW appears to be at the structural lattice level, changing from a C14 Laves phase and a bcc-type structure to two different bcc phases.

High-entropy alloys (HEAs), by definition, are materials composed of multiple principal metallic elements and are known to exhibit complex phase formation behavior, with intermetallic compounds such as Laves phases commonly emerging due to significant binary mixing enthalpy between the elements. Based on empirical HEA calculation principles, it would be reasonable to expect both CeFeTaVW and CrFeTiVW systems to form either a single bcc-type-structure solid solution or a bcc matrix accompanied by intermetallic phases, as demonstrated in earlier HEA systems [29,30,31]. According to the criteria established by Yurchenko et al. [49], the simultaneous presence of an atomic size mismatch (δ) above 5% and an Allen electronegativity difference, which shows the tendency of an atom to attract a shared pair of electrons towards itself in a chemical bond, exceeding 7%, promotes Laves phase formation in HEAs. Both alloys studied here satisfy these conditions: the Δχ Allen electronegativity (~14% for CrFeTiVW and ~15% for CeFeTaVW) and an atomic size mismatch of 6% or greater strongly indicate the thermodynamic feasibility of intermetallic precipitation. The binary mixing enthalpies for both Ta and Ti are considerably negative (Fe-Ta = −15 kJ/mol and Fe-Ti = −17kJ/mol), which supports the formation of these binary phases. For CrFeTaVW, an Fe-Ta Laves intermetallic phase was observed embedded in the CeFeTaVW matrix; however, for CrFeTiVW, no Laves phase was observed. Instead, two distinct bcc-derived structures are observed.

However, a report by Wu et al. [50], which studied the addition of Fe to a V-Ti-Cr alloy, shows a body-centered tetragonal (bct) phase with an experimental diffractogram that is almost identical to that observed in Figure 7. The bct structure is described as a distorted variant of a bcc lattice characterized by unequal axial lengths. Such a tetragonal distortion is well documented in V-Ti-Cr systems designed for hydrogen storage [51,52,53,54,55], where the lattice strain promotes hydrogen accommodation. Taking these findings into account, the distorted lattice configuration identified in CrFeTiVW suggests that the alloy could also yield a bct structure, driven by the substantial lattice strain characteristic of HEAs. This distortion mechanism provides a plausible and interesting explanation for the experimental diffractogram of CrFeTiVW, which shows a microstructure with only one major phase. Nevertheless, further crystallographic investigation, particularly via high-resolution transmission electron microscopy and local structural probing, is required to elucidate the origin and stability of this bct phase fully.

## 4. Conclusions

This study presents the results of the simulation of the structural and thermal characterization of equiatomic CrFeTaVW and CrFeTiVW high-entropy alloys. The Monte Carlo (MC) simulations indicate lower Gibbs free energies than the Molecular Dynamics (MD) simulations, resulting in more stable configurations for both CrFeTaVW and CrFeTiVW high-entropy alloys. Nevertheless, the divergent trends observed in the chemical segregation behavior—MC predicts overall lower segregation values than MD for CrFeTaVW, but for CrFeTiVW, MC predicts higher segregation values relative to MD—highlight the need for further investigation and refinement of the models. The pair distribution functions reveal noticeable tungsten clustering, evidenced by enhanced W–W bonding, in the CrFeTiVW system, whereas such segregation is less pronounced in CrFeTaVW. SEM and EDS images display comparable microstructures, while XRD shows a bcc-type structure with a Laves phase for CrFeTaVW and different bcc-type structures for CrFeTiVW. It is worth noting the presence of tungsten clusters, predicted by pair distribution function simulations, which might contribute to the XRD bcc-type peaks observed at 2θ = 40°.

Thermal diffusivity of the CrFeTaVW and CrFeTiVW alloys showed a slight increase with temperature, attributed to lattice expansion, which lengthens the phonon mean free path as temperature rises. Both entropy alloy systems displayed comparable results, as tantalum and titanium have nearly identical atomic radii (0.14 nm and 0.143 nm, respectively); their substitution does not significantly disturb the lattice or, therefore, the thermal diffusivity. Even so, the thermal diffusivity values for both alloys remain far below those of CuCrZr and W and fall within the range of 3 to 7 (mm^2^/s).

The results indicate that the CrFeTaVW and CrFeTiVW high-entropy alloys are promising for nuclear fusion applications, as their microstructures are simple and stable at high temperatures. Future work may include alloy composition optimization, evaluation of irradiation resistance, and study of mechanical properties.

## Figures and Tables

**Table 1:** Thermodynamic calculations for CrFeTaVW and CrFeTiVW high-entropy alloys.

Parameter	CrFeTaVW	CrFeTiVW
Δ*H*_mix_	−6.4 kJ/mol	−6.7 kJ/mol
Δ*S*_mix_	13.4 J/(K.mol)	13.4 J/(K.mol)
δ	5.4	6.1
VEC	6	5.8

## Data Availability

The original contributions presented in this study are included in the article/Supplementary Material. Further inquiries can be directed to the corresponding author.

## Supplementary Materials

The following supporting information can be downloaded at: https://www.mdpi.com/article/10.3390/ma19050987/s1, The LAMMPS input scripts will be shared.
